# Glass Transition and Re-Crystallization Phenomena of Frozen Materials and Their Effect on Frozen Food Quality

**DOI:** 10.3390/foods10020447

**Published:** 2021-02-18

**Authors:** Yrjö H. Roos

**Affiliations:** School of Food and Nutritional Sciences, University College Cork, T12 YK8AF Cork, Ireland; yrjo.roos@ucc.ie

**Keywords:** crystallization, freezing, glass transition, state diagrams, food quality

## Abstract

Noncrystalline, freeze-concentrated structures are formed during food freezing. Such freeze-concentrated food materials often exhibit crystallization and recrystallization phenomena which can be related to the state of solutes and water. State diagrams are important tools in mapping the physical state and time-dependent properties of frozen materials at various storage temperatures. Transition of simple solutions, such as sucrose, can be used to describe vitrification and ice melting in freeze-concentrated materials. A maximally freeze-concentrated material often shows glass transition at T_g_′. Ice melting occurs at temperatures above T_m_′ These transitions at temperatures above T_m_′ can be used to estimate crystallization and recrystallization phenomena and their rates in frozen foods. Furthermore, frozen food deterioration accelerates above T_m_′ and particularly as a result of temperature fluctuations during frozen food distribution and storage.

## 1. Introduction

Food freezing is widely utilized as a food preservation technology, and it has its origins in cold climates, which allow for natural freezing and subsequent storage during winter seasons. Even early attempts for the development of food freezing technologies suffered from the slow freezing of water and development of large ice crystals [1]. An industrial development of food freezing since 1924 was advanced by the inventions of Clarence Birdseye to freeze food more quickly inside a package between metal belts or plates [1]. Although the technology for industrial food freezing was invented, there was a clear need to improve product quality and to avoid attempts to preserve low-quality foods. Despite existing limitations to a full understanding of food freezing, the modern frozen food industry has grown to a substantial economic contributor with a market value around 300 billion US dollars in 2020.

Food freezing provides an excellent food preservation technology for a wide range of applications. Obviously, the freezing of animal products, vegetables, berries and fruits is widely accepted and used by the food industry as well as by individual households and consumers. Likewise, formulated foods, such as ice cream, can be preserved by freezing and even consumed directly from the frozen state. The large range of food compositions and food components with significantly different physicochemical properties often challenge the frozen food industry, that is, the microbial quality may remain high, but food materials may change both chemically and physically quite differently during freezing and frozen storage [2].

The main food preserving factors in food freezing are the dehydration of food solids because of the separation of water into dispersed ice crystals and a low temperature. A frozen food structure that is often dependent on the extent of freezing is temperature-dependent. This is because the freezing of water in biological materials occurs over a wide, composition-dependent temperature range. The freezing temperature depression of water in solutions is a well-established physicochemical property dictated by the fact that the chemical potential (μ) of a solvent at an equilibrium between two phases of the same substance must be equal. Typically, increasing molecular interactions decrease the chemical potential of solutions with a resultant freezing temperature depression [3].

A glass transition is a property of a nonequilibrium material that occurs as a reversible transition between solid-like (glassy) and liquid-like (rubbery, leathery, syrupy) states of an amorphous (disordered) material. Foods in their frozen state contain ice crystals imbedded in a noncrystalline, amorphous, unfrozen fluid (syrup). This is typically because sugars or a mix of multiple solutes in the aqueous phase often show delayed or inhibited solute crystallization. At a low temperature, solutes in the absence of solute crystallization become highly freeze-concentrated, and such freeze-concentrated solutes with some unfrozen water vitrify without crystallization as temperature is sufficiently lowered [4,5,6,7]. There are three principal requirements for the frozen food industry: (a) understanding food composition and its effects on food physicochemical properties during freezing; (b) understanding the impact of frozen food storage and distribution on frozen food stability and properties to retain frozen food quality; and (c) properties of food materials in thawing, including quality of frozen-consumed desserts. The present review highlights factors leading to the formation of noncrystalline, freeze-concentrated structures in food freezing and the physicochemical properties of frozen materials with noncrystalline phases, including solute and solvent recrystallization phenomena affecting frozen food characteristics and quality.

## 2. Freezing and Glass Transition

**Figure 1:**
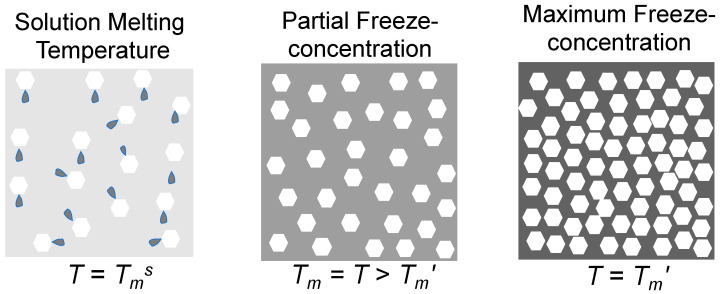
Schematic presentation of ice formation as affected by temperature, T. The equilibrium melting temperature for an unfrozen solution occurs at T_m_^s^. Equilibrium quantity of ice occurs at T_m_ during partial freeze concentration and maximum freeze concentration occurs at T_m_′.

It is interesting to investigate factors that control ice formation and the maximum freeze concentration in food-related aqueous materials. Sugars of high water solubility provide excellent models for such studies as sugar crystallization may be avoided despite the freeze concentration being above eutectic concentration (supersaturation). The freezing of water in solutions is related to the chemical potential, μ, which must be the same in solid and liquid states of water at the equilibrium freezing/melting temperature, T_m_. T_m_ is the highest temperature at which ice may exist during the rewarming of a frozen solution, i.e., the last ice crystals dissolve at T_m_. Ice in solutions and foods are formed as crystals that are made of water, and ice must have a lower chemical potential than water in the liquid state. The extent of freezing at normal frozen storage conditions of foods is primarily controlled by small solutes, such as sugars and dissociated salts. In other words, a temperature, T_m_, can be found where ice and unfrozen water have the same chemical potential. Quite often foods are not maximally freeze-concentrated, and therefore at a typical food storage temperature (T), T_m_ of ice surrounded by the freeze-concentrated unfrozen phase must be equal to T, and the amount of ice also varies with temperature to satisfy μ_solid_ = μ_liquid_ (Figure 1). Typical chemical, physical, biological, and other detrimental changes during frozen food storage occur within a partially freeze-concentrated phase [2].

Sugars are well-known glass formers, as was described by the early work of Parks and co-workers [8]. The evidence for a combined lactose–sucrose glass in ice cream was discussed by White and Cakebread [9] prior to the understanding of the amorphous structures of freeze-concentrated sugar solutions [10]. One of the arguments of White and Cakebread [9] as evidence for the presence of amorphous (noncrystalline) lactose, was that studies of Nickerson [11] indicated that lactose was stable below −23 °C, but crystallization often occurred at higher temperatures at a temperature-dependent rate. The studies of Bellows and King [4] explained the formation of a concentrated amorphous solute phase in sugar solutions with imbedded ice crystals. Furthermore, the studies of Levine and Slade [5], Roos [6] and Roos and Karel [12,13,14] provided new understanding of physicochemical properties of frozen sugar solutions and food materials. A freeze-concentrated sugar solution can now be described as an unfrozen material with supercooled, freeze-concentrated, rubbery or glassy continuous sugar-unfrozen water phase with embedded ice crystals. Freeze-concentrated materials exhibit two uncoupled transitions: (i) glass transition of a freeze-concentrated, unfrozen phase (reversible state transition); and (ii) ice formation (crystallization) and melting (phase transition). During freezing, glass transition results in a kinetic inhibition of translational mobility within the glass formers phase, while ice formation and melting follow thermodynamic reasons. Rewarming of a frozen material to above glass transition of its unfrozen phase allows for the reappearance of translational mobility and the associated physicochemical processes.

Biological and food materials often exhibit numerous phases in a frozen structure. The major phases include ice, hydrated macromolecules (oligosaccharides, polysaccharides, and proteins), freeze-concentrated solutes and ions, and crystallized or liquid lipids in a separate hydrophobic phase. Obviously, a structure of a particular frozen food is dependent on the type of the food and its physicochemical history. As water is the crystallizing solvent in frozen foods, ice formation results in a concentration of all dissolved substances and a partial separation of water from hydrated food components (dehydration). Simple aqueous solutions of highly soluble components, such as sugars, are often studied as models of food freezing to explain various thermal phenomena of nonequilibrium, amorphous states of freeze-concentrated solutes, and unfrozen water [5,12,13,14]. Unfortunately, some authors have presented quite confusing and physicochemically unrealistic theories for various changes in food-related materials at freezing conditions [15,16]. For example, a rapid cooling in such studies as well as partial melting and ice recrystallization during experimental procedures can result in variations in ice quantity and solution glass transition behavior. Indeed, a rapid cooling during the freezing of a sugar solution may result only in a partial freeze concentration of the solution and resultant inclusions of partially freeze-concentrated but vitrified sugar solution within ice crystals [15]. On the other hand, careful freezing and annealing as reported by Roos and Karel [12,13,14] can be used to avoid such artefacts in experimental studies. Therefore, careful annealing to control the thermal history of samples must be used to achieve maximum ice formation without misleading artefacts of multiple transitions. Such artefacts are often caused by a time-dependent variation of multiple phenomena, such as freeze concentration, vitrification and devitrification, recrystallization, and ice formation. It should be noted that water molecules in a maximally freeze-concentrated solute phase retain high mobility [17], and unfrozen water should never be referred to as “unfreezable” water.

**Figure 2:**
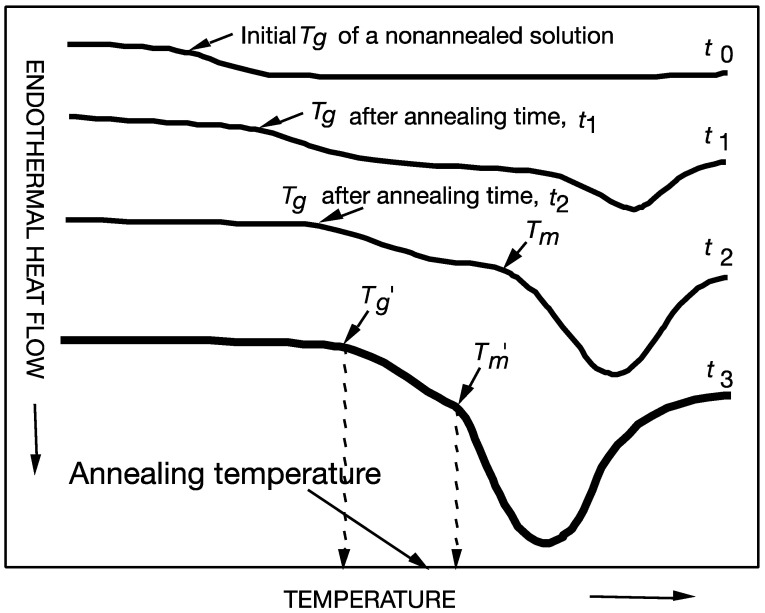
Ice formation in aqueous solutions during isothermal holding using differential scanning calorimetry (DSC) at various temperatures to enhance ice formation (annealing). A maximally freeze-concentrated state can be achieved during isothermal holding slightly below the onset of ice melting temperature, T_m_′. An increase in ice content is shown to increase the freeze concentration and glass transition of the freeze-concentrated solution until maximum ice formation is achieved after annealing for t_3_. Freeze concentration increases during annealing and results in a higher glass transition temperature, T_g_, as plasticizing water crystallizes into an ice phase.

**Figure 3:**
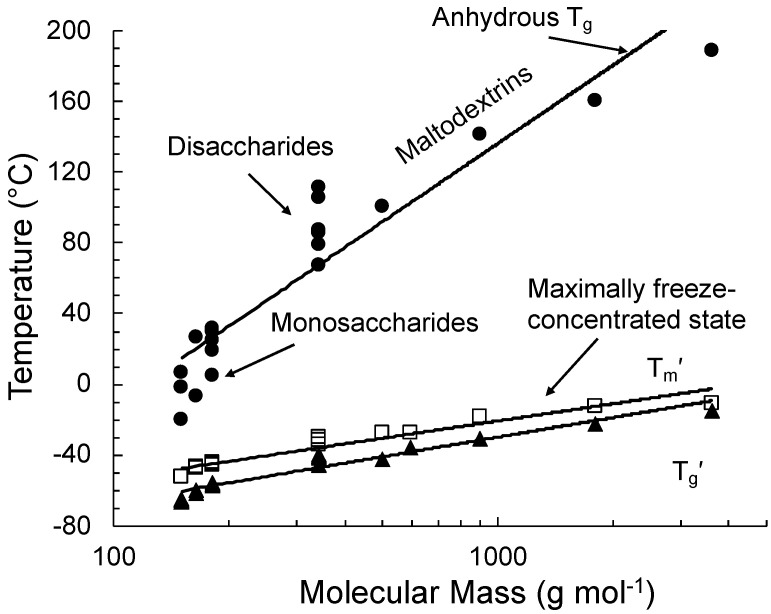
State transitions of various carbohydrate food components and their dependence on molecular mass in binary aqueous solutions (data from Roos [18,19]).

Glass transitions of freeze-concentrated solutions often occur at low temperatures and well below the normal storage and distribution temperatures of frozen foods. Roos and Karel [12] used sucrose solutions as models to explain time-dependent ice formation during freezing and annealing treatments in the determination of thermal phenomena in a maximally freeze-concentrated material. Most food materials show no solute crystallization during freezing, and a glass transition of dissolved solutes with a quantity of unfrozen water as a plasticizer takes place during freezing [5,12]. Differential scanning calorimetry (DSC) is often used to record the onset temperatures for glass transition (T_g_′) and for ice melting (T_m_′) during the heating of a maximally freeze-concentrated material. Maximum ice formation produces a maximally freeze-concentrated state of solutes as described in Figure 1 and Figure 2. The glass transition occurs as an endothermic step in heat flow, and the onset temperature of the maximally freeze-concentrated unfrozen phase is taken as T_g_′. A single solute may concentrate in unfrozen water, and such an unfrozen phase forms a continuous matrix around ice crystals. During the heating of a maximally freeze-concentrated solution, the onset of an ice melting endotherm is found at T_m_′. It should be noted that the glass transition occurs over a temperature range as a separate transition from ice melting. The glass transition may not complete as ice melting occurs above T_m_′, and T_g_′ and T_m_′ may also coincide at the same temperature [12,13,14]. The solute concentration in the maximally freeze-concentrated, unfrozen phase is referred to as C_g_′, and its water content is given by W_g_′ (unfrozen but not unfreezable water). The glass transition temperatures for various sugars and carbohydrates as shown in Figure 3 can be found in Roos [18,19] and Roos and Drusch [20]. A comprehensive list of T_g_′, T_m_′, and T_m_ values for various foods was reported by Kumar et al. [2].

The glass transition of a maximally freeze-concentrated solution is the property of the solute that is plasticized by unfrozen water. The respective quantities of the solute and unfrozen water are referred to as C_g_′ and W_g_′, respectively. Roos and Karel [12] showed that noncrystalline sucrose that was plasticized by water corresponding to the maximally freeze-concentrated state showed no ice formation but rather a glass transition at the T_g_′ of sucrose. Roos and Karel [13] showed that the glass transition of maximally freeze-concentrated fructose and glucose solutions could also be reproduced in highly concentrated syrups with solute concentrations corresponding to the respective C_g_′ of these sugars. The study of Roos [18] investigated glass transitions of various sugars and sugar alcohols. The T_g_ and T_g_′ data included in Figure 3 were related to properties of individual sugars and carbohydrates in general while the freeze concentration of solutes occurred to approximately 80% by mass with 20% by mass unfrozen water in the maximally freeze-concentrated state.

**Figure 4:**
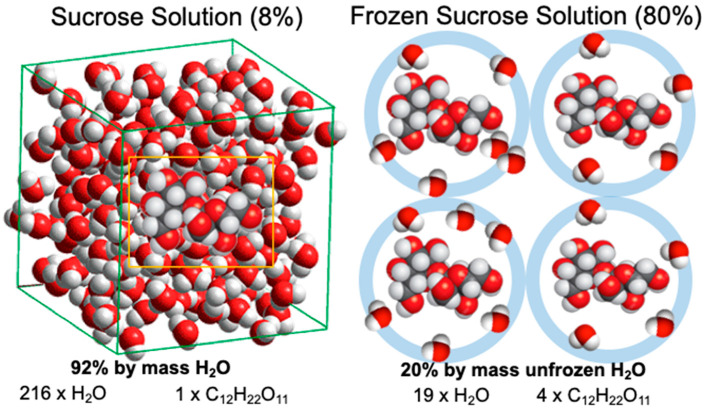
A schematic illustration of maximum freeze concentration in sucrose solutions. An 8% sucrose solution shows freeze concentration until 1 mol of sucrose molecules undergoes hydrogen bonding with approximately 4.75 mol water.

Roos and Karel [12] showed that in a maximally freeze-concentrated state, approximately 0.25 g of water/1 g of sucrose remained unfrozen. Their finding suggested that approximately 4.75 molecules of water were structured by hydrogen bonding around each sucrose molecule within a maximally freeze-concentrated solution (Figure 4). On the other hand, water molecules within a freeze-concentrated or glassy state of sugars show a high mobility [17], but ice formation ceases at T < T_m_′. Consequently, it can be assumed that chemical potentials of unfrozen water and ice at T_m_′ must be equal, but at temperatures T < T_m_′ a lower chemical potential of water molecules hydrogen-bonded to sucrose must inhibit ice formation. Furthermore, vitrification occurs below T_m_′ at temperatures below T_g_′. It is also important to note that vitrified partially freeze-concentrated solutions do not show ice formation below their respective glass transitions, as nucleation and crystal growth become kinetically inhibited and spatially limited in the glassy state [5,12].

## 3. State Diagrams

A simple state diagram shows transition temperatures and other physicochemical properties of hydrophilic glass formers against solids mass fraction. State diagrams are also known as supplemented phase diagrams, which have been published over the years for food components and food products [21,22,23,24,25,26]. Although state diagram data are incorrectly reported for single food products such as dried fruits, the reported transition temperatures apply rather to single glass formers or blends of miscible glass formers forming only part of the product solids.

**Figure 5:**
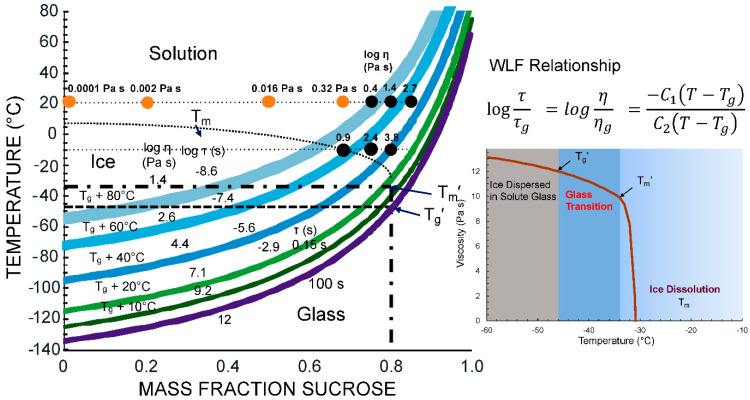
State diagram of sucrose showing physical state and its dependence on temperature and water content. Viscosity data from Longinotti and Corti [28] are shown by closed symbols. The Williams–Landel–Ferry (WLF) relationship [29] was used to estimate structural relaxation times, τ, and viscosity, η, above the calorimetric onset glass transition with corresponding τ_g_ = 100 s and η_g_ = 10^12^ Pa s [30]. Parallel curves indicating structural relaxation times with corresponding isoviscous states are shown for various T–T_g_ conditions. The effect glass transition of the maximally freeze-concentrated solution, T_g_′, and ice melting above the T_g_′ corresponding ice melting temperature, T_m_′, is shown in the inset diagram.

Figure 5 shows a state diagram with the expected relaxation time and corresponding viscosity data, including description of effects of T_g_′ and T_m_′ followed by equilibrium melting according to T_m_, for freeze-concentrated sucrose solutions. The relaxation time data emphasize the importance of kinetic limitations of crystallization phenomena in the vicinity of the glass transition and T_m_′. Furthermore, the dramatic effect of temperature on relaxation times and viscosity above T_m_′ can be explained by a highly temperature-dependent extent of the freeze concentration. State diagrams provide guidance for food freezing and frozen storage as they particularly identify the conditions required for enhanced frozen state stability [19,27].

Levine and Slade [5] emphasized the importance of glass transition-based cryostabilization technology. Cryostabilization refers to storage of food materials below T_m_′. Temperatures above T_m_′ show significant variations in unfrozen water content and therefore the increased rates of solute and solvent crystallization as well as other deteriorative changes. Simple state diagrams assume that the solid, glassy state is formed at temperatures below T_g_. The viscosity of the glassy state is assumed to exhibit values >10^12^ Pa s (Figure 5) while relaxations times above the T_g_ decrease exponentially, reflecting an increase in liquid-like characteristics as the translational mobility of glass-forming molecules appears at temperatures above the T_g_. The typical crystallization phenomena of foods, such as solute (hydrophilic components) and solvent (water) crystallization, are not expected to occur within a glassy material because of kinetic and spatial limitations. According to Kumar et al. [2], food materials show low to medium sensitivity to storage above T_g_′ (state transition sensitivity) while a severe sensitivity is found to temperatures above T_m_′ (phase transition sensitivity).

Foods typically show complex interactions of concentrated constituents in the freeze-concentrated state. However, the chemical potential of water across food components at a given temperature must have the same value, and water distribution can be assumed to occur according to its fractional affinity to each constituent. Such assumption allowed Roos and Potes [31] to construct a tertiary state diagram for a carbohydrate–protein–water system. They also could find amounts of unfrozen water distribution to carbohydrate and protein components.

## 4. Solute Crystallization

Solute crystallization in frozen foods may be regarded as a harmful, spontaneous transition of noncrystalline solutes as they exhibit a thermodynamic driving force towards their equilibrium, crystalline state. Solute crystallization is typical of poorly soluble food components, such as lactose. As normal temperatures in frozen food storage are below −18 °C, solute crystallization is limited to food components with T_m_′ below −18 °C.

Nickerson [11] pointed out that lactose in ice cream could exist as a lactose glass, which was known to be the case in dry milk. The glassy state of lactose was recognized by White and Cakebread [9] as the main factor controlling lactose crystallization that is perceived as “sandiness” in ice cream, i.e., particle size of lactose crystals increases to above a 20–50 μm size limit. Such coarse crystals are sensed by a human tongue as gritty particles. Solute crystallization in frozen foods may occur above T_g_′ as a result of supersaturation at a temperature below the eutectic temperature (solubility limit). Lactose exhibits a poor water solubility at low temperatures [32,33], and therefore the crystallization of lactose within a freeze-concentrated lactose-containing food material, such as ice cream, is likely. The rate of lactose crystallization is highly temperature-dependent and exhibits a maximum at a temperature where the rate becomes increased by supersaturation without significant decrease caused by increased viscosity at a lower temperature due to freeze concentration and decreasing temperature [12]. The crystallization of sugars at temperatures above T_g_′ can become inhibited in blends with several sugars and by using hydrocolloids to increase the viscosity of the freeze-concentrated, unfrozen phase.

## 5. Ice Recrystallization

Ice formation in foods during freezing results from a thermodynamic driving force towards nucleation and crystal growth at T < T_m_. A rapid freezing of foods is often considered essential in the manufacturing of high-quality frozen foods with a large number of small crystals. The initial size of ice crystals is determined by nucleation: Rapid cooling during freezing to a low nucleation temperature may be used to obtain a large number of nuclei. As a result, a large number of small crystals occur in a rapidly frozen food. Such small crystals may remain stable at T < T_m_′, but at temperatures above T_m_′, and particularly as a result of temperature fluctuations, ice recrystallization leads to increasing the ice crystal size during frozen food distribution and storage [2]

Ice formation at any concentration less than the mass fraction of solutes within a maximally freeze-concentrated solution, C_g_′, occurs according to the T_m_ curve shown in Figure 5. Ice formation ceases at T_m_′ as the chemical potential of water molecules undergoing hydrogen bonding to solute molecules becomes less than that of ice. Such an assumption explains a sudden increase in the heat capacity associated with T_m_′ of freeze-concentrated solutions during heating over the T_g_′ and T_m_′ temperature range [12]. On the other hand, the rapid cooling of solutions to temperatures below their unfrozen T_g_ may result in vitrification without ice formation. Such quench-cooled materials may not exhibit ice formation below the T_g_ as ice formation is kinetically and spatially inhibited within a glassy material. A quench-cooled material shows ice formation during reheating to above T_g_. Such ice formation occurring during the reheating of a vitrified solution is known as devitrification [18]. Ice formation during the rewarming of food may also be referred to as irruptive recrystallization [2].

Recrystallization may be considered as a phenomenon occurring subsequently to crystal growth stage during a crystallization process where the shape and size of ice crystals are modified [19]. Ice recrystallization in frozen foods is likely to occur at temperatures above T_m_′ with rates increasing with temperature due to dissolving of ice crystals and a higher unfrozen water content. The main recrystallization types are known as isomass, migration, and accretive recrystallization [2,19], which are described as follows:

Isomass recrystallization— Isomass recrystallization refers to a change of a crystal without a change in mass. This is often seen as the rounding of crystals, as sharp edges exhibit a higher chemical potential of ice than round corners. In other words, crystal shape is rounded to minimize the free energy of water molecules.
Migratory recrystallization— Migratory recrystallization is also known as Ostwald ripening. Migratory recrystallization occurs between several crystals of varying sizes as larger crystals size increases at the expense of smaller crystals.
Accretive recrystallization— Accretion involves two or more crystals as they merge into a single, larger crystal.

Recrystallization rates are strongly dependent on temperature and diffusion above a rate controlling T–T_g_ value. It can be assumed that dilution above T_m_′ accelerates the recrystallization processes as viscosity becomes dependent on both temperature and dilution (Figure 5). An increase in ice crystal size is generally expected to result in icy food materials and a significantly lower food quality. Furthermore, recrystallization leads to the damage of cellular structures and changes in food texture during thawing [2,23].

Besides recrystallization phenomena occurring depending on material composition, temperature, and physicochemical surroundings of crystals at a constant temperature above T_m_′, frozen food quality is likely to deteriorate during food distribution and storage. Temperature fluctuations resulting from normal temperature variation during frozen food storage may significantly accelerate losses of quality [2,34].

## 6. Conclusions

Ice formation in frozen food materials results in concomitant freeze concentration of solutes. At a sufficiently low temperature, an unfrozen phase with solutes and unfrozen water may vitrify. The onset temperature of ice melting of the maximally freeze-concentrated material, T_m_′, can be related to solute and solvent (water) crystallization and recrystallization during food storage. Solute crystallization decreases the quality of frozen-consumed foods, such as ice-cream. Recrystallization of ice may occur through various mechanisms as the viscosity of the unfrozen phase of a freeze-concentrated phase above T_m_′ becomes temperature and dilution-dependent. Glass transition and ice-melting data are required for key food ingredients in order for the crystallization phenomena to be controlled during freezing, frozen food distribution, and frozen storage. Such data on glass transitions in frozen foods have significantly contributed to improvements in the manufacturing and storage of frozen foods, including ice cream and frozen desserts. Obvious improvements in manufacturing and product stabilization technologies have occurred through the selection of ingredients with known transition properties, control of ice crystal size, and reduced recrystallization during food distribution and storage.
